# Babesiosis in Essex, UK: monitoring and learning lessons from a novel disease outbreak

**DOI:** 10.1186/s13071-018-2718-7

**Published:** 2018-03-20

**Authors:** Ian Wright

**Affiliations:** The Mount Veterinary Practice, 1 Harris Str, Fleetwood Lancs, FY7 6QX UK

**Keywords:** *Babesia*, Babesiosis, *Dermacentor*, Ticks, Surveillance

## Abstract

Canine babesiosis is a parasitic disease caused by apicomplexan protozoa of the genus *Babesia*, with *Babesia canis* being a pathogenic and widespread species in mainland Europe. The United Kingdom has thought to have been free of endemic *B. canis* infection, despite its vector, *Dermacentor reticulatus* being present in endemic foci. The winter of 2015/2016 saw the establishment of the first recording of a known endemic foci of *B. canis* in the UK. Since this outbreak in Harlow and subsequent cases in Romford later in 2016, information has been gathered regarding the population of *Dermacentor* ticks in Harlow and awareness of the disease promoted among Veterinary professionals and pet owners. This letter describes what is known about the two clusters of cases seen in 2016 and the distribution of *D. reticulatus* in the UK. A further untraveled case in the UK in 2017 close proximity to the 2016 cases is also described, as well as the lessons this outbreak brings in terms of managing other vector-borne disease.

## Letter to the editor

Canine babesiosis is a tick-borne disease caused by species of intraerythrocytic apicomplexan protozoa belonging to the genus *Babesia* [1]. Infection occurs when the parasite is transmitted in the saliva of a feeding tick, blood transfusion, and in the case of *Babesia gibsoni*, through dog bites [2]. Although a number of *Babesia* spp. are present throughout Europe, *Babesia canis* is the most common [3], being highly prevalent in many mainland European countries including France [4] and Portugal [5]. Infection can lead to red cell lysis and immune mediated disease, resulting in anaemia, icterus, lymphadenopathy, pyrexia, secondary renal and hepatic disease, and in severe cases, death [1]. Its distribution is closely associated with its vector, *Dermacentor reticulatus* [3]. Although foci of *Dermacentor* ticks had been known to be present in the United Kingdom (UK) [6], and a fatal case of babesiosis caused by *B. canis* had been recorded in an untraveled dog in the UK, it was not thought to be endemic [7]. The presence of *D. reticulatus* endemic foci in the country however, in the face of increased pet travel and importation [8], made the UK susceptible to foci of *B. canis* establishing. This was found to have occurred in the winter of 2015/2016 when *B. canis* was reported in four untraveled dogs in Harlow, Essex [9], followed by subsequent cases in 2016 [10] and 2017 [11].

Between November 2015 and February 2016, there were four confirmed cases of *B. canis* in untraveled dogs from Harlow, Essex [9]. The first case was presented to the Royal Veterinary College and was typical. There was a three-day history of anorexia and haemoglobinuria, moderate anaemia and thrombocytopaenia. *Babesia canis* was confirmed by the identification of piroplasms on blood smears from peripheral ear veins and by polymerase chain reaction (PCR) [12]. *Dermacentor* ticks recovered from infected dogs were confirmed by PCR to be *D. reticulatus* infected with *B. canis* [13]. To investigate this outbreak, a tick survey was carried out in the area where all four dogs had been regularly walked, with the aim of establishing if an active population of infected *Dermacentor* ticks was present. Eight female and nine male ticks were collected from a field in Harlow, morphologically identified as *D. reticulatus*. This was confirmed by DNA barcoding and 14 of the ticks were found to be positive for *B. canis* by PCR [14]. This established the presence of an infected population of *D. reticulatus* ticks in the proximity of the infected dogs and likely the source of infection. This area is a popular dog walking route and local authorities acted quickly to erect a barrier to discourage access to the field and signs erected, educating owners as to the risk of *B. canis* in the area [14]. These preventative measures in combination with promoting tick preventative measures and vigilance to dog owners appeared to be effective as there were no further cases. *Dermacentor reticulatus* however, is typically a tick active during the autumn and winter months [15] and while spring activity is recognised, there was the possibility of a cessation in tick activity and cases until the following autumn.

Two new cases of *Babesia* were confirmed in August 2016 in dogs from Romford, Essex. The dogs had no history of foreign travel and no known connection with the *Babesia* cases in Harlow. An 11-year-old Labrador was brought to the Best Friends Veterinary Group in Romford as an emergency but was dead on arrival. When asked about ticks, the owner reported finding two on the dog a fortnight previously. Blood testing revealed *Babesia* spp. [10]. In the second case, a male Labrador was brought to the same surgery after his owner had found and attempted to remove two ticks from his body. After 12 days the dog returned to the practice with high fever, lethargy and anaemia. Blood smears were positive for *Babesia* spp. [10]. After initial treatment with fluids and clindamycin, followed by imidocarb, which was received after three days, the dog went on to make a full recovery.

The latest case of babesiosis in an untraveled UK dog was identified when an eight-year-old Staffordshire bull terrier presented at the Walton Lodge Veterinary Group surgery in Ware, Hertfordshire in late August 2017 after the animal’s owners noticed blood in its urine [11]. *Babesia* infection was confirmed but not speciated by piroplasm detection in peripheral blood smears and a full recovery made after treatment with imidocarb.

Field surveys conducted in England and Wales during 2009–2016 confirmed the presence of *D. reticulatus* in four main areas in England and Wales. These are western Wales, north Devon, south Devon and Essex [15]. The population sites in western Wales were largely dune habitats, demonstrating the niche ecosystems that *D. reticulatus* often inhabit, but ticks were also found on cattle and in the proximity of a camp site inland, demonstrating the small foci and adaptability that *D. reticulatus* populations can exhibit [15]. Cattle are likely to be important hosts for adult stages of *D. reticulatus* and rodents for nymphs but the possible association with rabbits suggests that this host species may also play an important role in feeding immature stages [16]. The predominance of sand dune habitats for *D. reticulatus* in the UK is not demonstrated in other European populations [15]. *Dermacentor reticulatus* ticks appear to quest on the periphery of habitats however, where hosts may be passing, particularly paths regularly used by dogs [15]. They may also easily survive on managed meadows and pastures, making contact in a variety of rural locales possible. The Essex population so far is unique in the UK, in not being coastal or in the proximity of dune systems, suggesting that ticks may have been moved there by pets, livestock or vehicles [17]. This makes further spread in Essex more likely, as well as possibly elsewhere in the UK where *Dermacentor* ticks have been identified on untraveled dogs [18].

Since the Pet Travel Scheme (PETS) was relaxed in 2012, pet travel has increased year on year, increasing from 140,000 dogs travelling from the UK in 2012 to 164,800 in 2015 [8]. This increase in pet travel has occurred at a time of increased human migration and climate change, providing favourable conditions for the rapid spread of parasitic diseases and their vectors. In addition, the importation of dogs from other countries are also increasing the likelihood of novel parasites being introduced [8]. These factors can allow the establishment of vector-borne disease in a number of ways.

- **Introduction of parasites into existing vector populations “vectors waiting for a disease”.** *Dermacentor reticulatus* and *Ixodes* spp. ticks are already endemic in the UK [6] and are potential vectors of *Babesia canis* and tick-borne encephalitis, respectively [19].
- **Introduction of parasites into areas where vectors have spread.** *Thelazia callipaeda* has spread North across Europe in the wake of its *Phortica* spp. fruit fly vector. While climate, vehicle transport and wind dispersal are factors in the distribution of the fruit fly, pet movements are rapidly leading to the spread of *T. callipaeda* to new countries as well [19]. The spread of *D. reticulatus* within and between countries similarly provides a framework through which *B. canis* could spread and establish.
- **Introduction of vectors and parasites together.** *Rhipicephalus* have arrived in the UK on travelled and imported dogs, potentially exposing UK dogs to infections that the tick carries such as *Ehrlichia canis*, without the tick being endemic [17, 19].
- **Introduction of vector-borne parasites that are then transmitted in the absence of the vector.** *Leishmania infantum* has established in countries such as Canada with no sand fly vector purely through venereal transmission without a vector being present [19].

Veterinary professionals must therefore be aware of exotic pathogens being present in pets entering the UK from abroad and be prepared to give accurate parasite control advice to owners planning to travel with their pets. This recent outbreak of *B. canis* in the south-east of England demonstrates the benefits of a rapid and coordinated approach to exotic disease control. Whenever a new vector-borne disease emerges in a new location there is uncertainty as to whether infected pets have genuinely not travelled abroad and whether infection will persist in vector populations. In the case of *B. canis*, transovarian transmission circulating in vector populations may increase this likelihood. The initial outbreak of babesiosis in untraveled dogs from Harlow was rapidly brought to the attention of veterinary professionals and the public. It was confirmed to be of UK origin by the thorough work of a local veterinary practice, linking the first 3 cases [9]. This was followed by confirmation of a local population of infected *D. reticulatus* ticks [13]. Rapid confirmation that cases are linked to endemic foci of infected vectors is crucial to predict the likelihood of further outbreaks and keep vigilance high among local vets and pet owners. This vigilance led to the rapid diagnosis and treatment of two further cases months later and their full recovery. Surveillance of other groups of potential vectors is important to be both aware of areas where the parasite may be likely to spread, and for the potential for endemic vector foci to change dynamically. The possibility that *D. reticulatus* ticks have spread to Essex via animal or vehicular movements, means that promoting tick control and awareness among pet owners is also vital to limit further spread of the vector. Warning signs to pet owners and physical barriers to prevent access of vectors to potential hosts is useful in the face of a tick vector and/or disease outbreak. Removal of tick habitats and spraying with insecticides may succeed in eliminating a known tick and/or tick-borne disease focus but this can be difficult or impractical in ecologically sensitive areas or popular tourist areas.

## Abbreviations

PETS: Pet Travel Scheme
